# Consideration of compound drivers and impacts in the disaster risk reduction cycle

**DOI:** 10.1016/j.isci.2023.106030

**Published:** 2023-01-25

**Authors:** Bart J.J.M. van den Hurk, Christopher J. White, Alexandre M. Ramos, Philip J. Ward, Olivia Martius, Indiana Olbert, Kathryn Roscoe, Henrique M.D. Goulart, Jakob Zscheischler

**Affiliations:** 1 Deltares, Delft, the Netherlands; 2 Institute for Environmental Studies, VU University Amsterdam, the Netherlands; 3 University of Strathclyde, Glasgow, UK; 4 Institute of Meteorology and Climate Research, Karlsruhe Institute of Technology, Karlsruhe, Germany; 5 Instituto Dom Luiz (IDL), Faculdade de Ciências, Universidade de Lisboa, Portugal; 6 Institute of Geography and Oeschger Centre for Climate Change Research, University of Bern, Bern; 7 National University of Ireland, Galway, Ireland; 8 Department of Computational Hydrosystems, Helmholtz Centre for Environmental Research – UFZ, Leipzig, Germany

**Keywords:** Earth sciences, Social sciences, Decision science

## Abstract

Consideration of compound drivers and impacts are often missing from applications within the Disaster Risk Reduction (DRR) cycle, leading to poorer understanding of risk and benefits of actions. The need to include compound considerations is known, but lack of guidance is prohibiting practitioners from including these considerations. This article makes a step toward practitioner guidance by providing examples where consideration of compound drivers, hazards, and impacts may affect different application domains within disaster risk management. We discern five DRR categories and provide illustrative examples of studies that highlight the role of “compound thinking” in early warning, emergency response, infrastructure management, long-term planning, and capacity building. We conclude with a number of common elements that may contribute to the development of practical guidelines to develop appropriate applications for risk management.

## Graphical abstract

**Figure:**
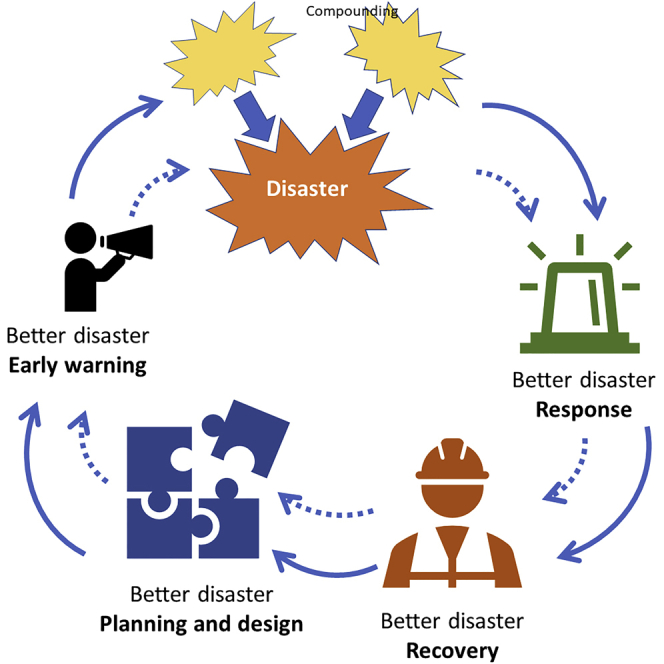

## Introduction

Human and ecological disasters induced by extreme weather-related events are usually governed by multiple compounding features, leading to hazardous conditions, or following complex pathways from hazard to impacts. Disaster management can be structured in different groups of applications, such as early warning systems, infrastructure improvement projects, or damage recovery programs. Within these applications—ranked in the disaster risk reduction cycle—important compound driver and impact considerations are often neglected.^1^ Compound drivers and hazards refer to coinciding hydrometeorological forces that govern impacts and risk, such as episodes with compounding extreme temperature, winds or high surge, excessive rainfall or long-term droughts, or extremely high or low river discharge.^2^ Multiple compounding impacts can be generated by a single hydrometeorological hazard (such as a strong atmospheric circulation inducing simultaneous or subsequent combinations of floods, infrastructure damage, heat waves, or wildfires), and the way consecutive hazards accumulate over space or time can strongly affect the societal impacts.^3^ Large or small changes in the timing, location, or intensity of one or more of the combinations of hydrometeorological forces or their consequences may lead to a considerable change in the resulting impact. When high runoff coincides with storm surge, high tide, and/or high surge waves, flooding levels can be considerably higher.^4^ The timing of droughts and heat waves determines whether an ecosystem increases its carbon uptake or becomes a carbon source.^5^ The joint occurrence of heat waves and droughts is a major source of risk to several socioeconomic activities.^6^ Neglecting compound driver and impact considerations can lead to an underestimation of risk, inappropriate emergency response, or poor adjustment of multiple actions within the disaster risk management cycle.

The need to assess compound hazards, drivers, and impacts has been recognized since the 1970s^7^ and has recently been documented in a collection of case studies, reviews, and assessment reports.^1^^,^^8^^,^^9^

Compound events can be classified in multiple ways. Zscheischler et al. (2020)^10^ proposed a clustering typology from a climatic hazard (or impact-driver) perspective, whereas Raymond et al. (2020)^11^ considered the impact of compound events on the risk profile in different societal sectors (food, water, health, infrastructure, and insurance) operating at different governance scales. This classification helps the scientific and societal discourse on environmental risk management in the context of a changing climate,^12^ but does not provide application-specific guidance on when and how compound considerations should be included in the disaster risk reduction cycle. Further guidance is needed to include compound considerations in practice.^13^

For some practitioners operating in risk management disciplines, the notion of concurrent impact-drivers is well established and implemented in their portfolio of activities. However, in a changing climate and societal setting the practice of disaster risk reduction requires a continuous adjustment and upgrade of existing practices and applications. The increasing array of hazardous events that contain a surprising and unprecedented combination of impact-drivers contains a call for action to adjust the current environmental risk assessment and management procedures by incorporating the trends in the climate statistics and in the insights of the potential combination of high-impact drivers.

In this article we make a step toward providing practitioners inspiration for this upgrade process. We identify and discuss several environmental application domains within the disaster risk reduction cycle for which consideration of compound features improves the risk reduction benefits of different actions. Compound thinking refers to the awareness, consideration, and improved representation of compound impact-drivers (hazards such as surge, heavy rainfall, extreme temperatures, etc.) as well as compound impacts (accumulation of societal or ecological impacts over space and time). This article provides examples of this compound thinking, and illustrates how it did or could improve management of risks where weather and climate features play a prominent role. This application-oriented view on compound events provides a complementary perspective to increase our grip on strategies to improve their monitoring, modeling, and interpretation.

In the next subsection we describe a rough classification of environmental application domains ranked in different stages of disaster and climate risk management. We will discuss the role of compound events in each of the application clusters. The underlying literature, although not comprehensive, serves the purpose to illustrate potential ways forward by putting the identification and management of compound events in the spotlight.

## Classification of application domains within the disaster risk reduction cycle

**Figure 1:**
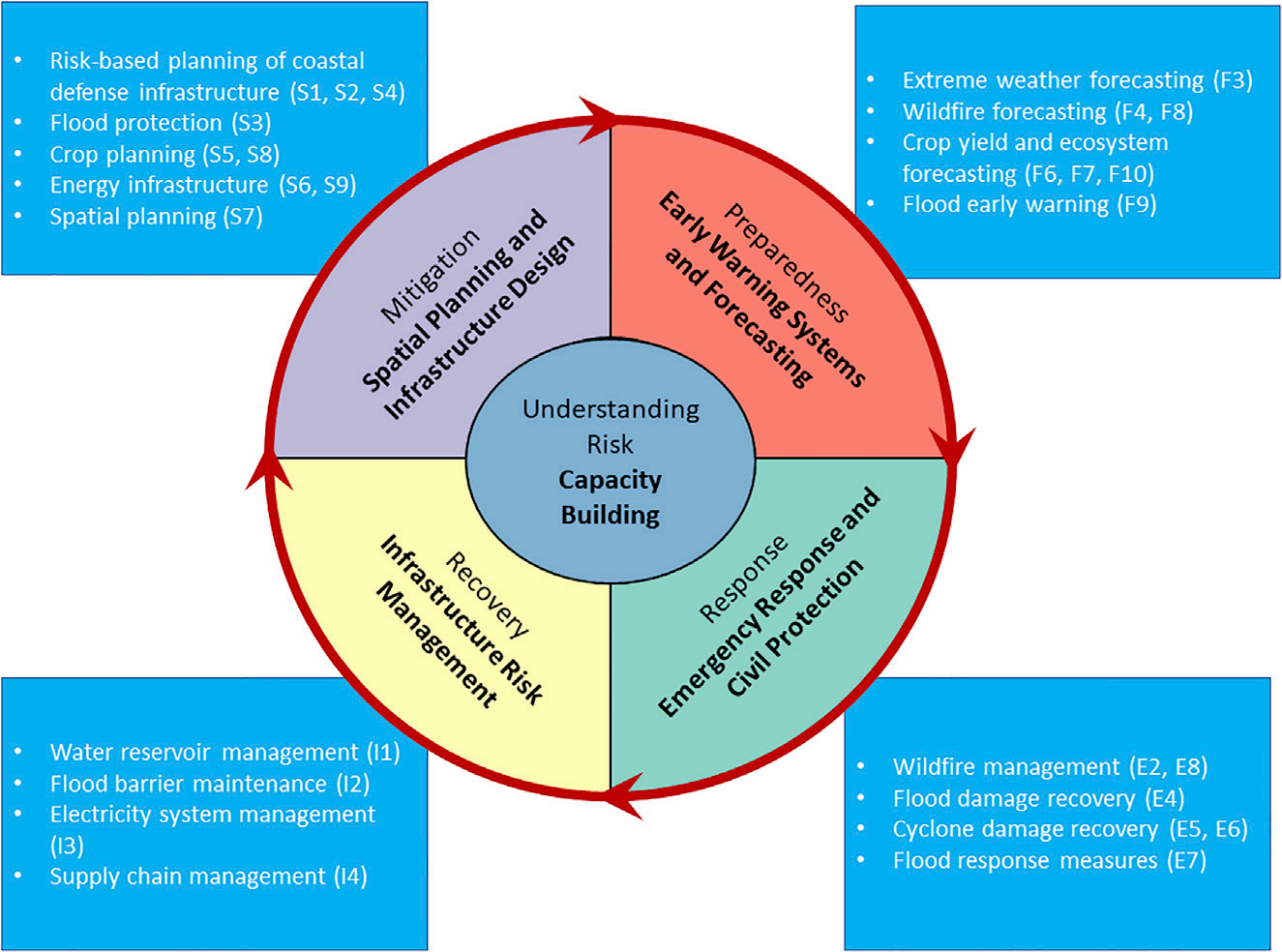
Grouping of application domains over various stages of the Disaster Risk Reduction cycle Note that “Infrastructure Risk Management” in practice covers multiple DRR stages, but is located in only one segment for graphical clarity. Examples of applications are listed per category, and cross-referenced to the case study overview in Table 1.

Environmental risk management increasingly involves applications that are designed to reduce disaster risks.^14^ These applications can be classified by the different phases in the Disaster Risk Reduction (DRR) cycle (preparation, mitigation, response, and recovery).^15^ These are adopted, for instance, in the main targets of the Sendai Framework for Disaster Risk Reduction^16^ and the European Union Civil Protection Mechanism.^17^ Here we discern the following compound event application domains, their association with the DRR stages, and their specific role in disaster risk management (see also Figure 1).

• *Early warning systems and forecasting* (“preparedness”): societal preparedness for environmental hazards relies on the ability to anticipate upcoming high-impact hazards. This includes the reliance on historic analyses, reliable observations, and physical models that are able to provide (probabilistic) forecasts of drivers, hazards, and their potential impacts;
• *Emergency response and civil protection* (“response”): the design of the emergency response system (including civil protection and material for emergency preparation, relief and recovery) relies on an adequate assessment of extreme compound events and their joint impacts;
• *Infrastructure risk management* (“preparedness,” “response,” and “recovery”): the management of infrastructure, the built environment, and the functioning of public and private institutions relies on the monitoring of complex impact-drivers and inventories of options to anticipate and mitigate impacts of hazards and to quickly restore operations and repair infrastructure;
• *Spatial planning and infrastructure design* (“mitigation”): the enhancement of societal resilience against risk relies on adequate spatial planning and infrastructure design contributing to prevention and mitigation of environmental impacts. This requires an appropriate representation of compounding drivers of impacts that are considered in this design process;
• *Capacity building* (“understanding risk”): includes capacity building with a solid theoretical and practical knowledge base necessary for the design of adequate monitoring and modeling concepts, training, and multi-disciplinary innovation.

The following section presents an illustrative review of each of these application domains, approached from the perspective of compounding impact-drivers and hazards retrieved from literature.

## The role of compound events in application domains

The typology of Zscheischler et al. (2020)^10^ provides some guidance on how considerations of compound events in risk management applications can be improved. *Preconditioned compound events* refer to the importance of antecedent conditions in explaining the severity of the impact following a hydrometeorological hazard, such as the dependence of flood severity on soil moisture saturation.^18^ *Multivariate compound events* need to be considered when multiple hazards jointly determine the impact, such as the co-occurrence of a strong surge and heavy rainfall in a coastal flooding scenario.^19^ *Temporally compounding events* include situations in which multiple hazards subsequently lead to an impact in a given region, and these can be either sequences of similar hazards (such as series of tropical cyclones,^20^) or different types of events (such as a tropical cyclone followed by a heat wave^21^). *Spatially compounding events* concern multiple events affecting connected regions in a limited time window, such as the risk of simultaneous crop failure in multiple bread-basket regions.^22^

For many events this typology does leave room for interpretation of the most appropriate category, but it can give indications in what direction the disaster risk management applications need to incorporate compound events. It may emphasize the need for careful assessment of initial conditions while making event forecasts, consider high-impact events in adjacent regions, analyze climate statistics of correlated hazards, or design multi-purpose protection infrastructure. In the following we will elaborate a number of examples of compound events and their impacts found in literature, grouped by the application domains shown in Figure 1. For each example we will allocate the most appropriate compound event type following Zscheischler et al. (2020),^10^ and revisit the distribution of these types over the application domains (see Table 1).

**Table 1:** List of cited case studies ranked per Disaster Risk Reduction application domain

Nr	Case study	Compound event typology	Compounding features	Application	Citation
[F]	*Early warning systems and forecasting*				
[1]	Coastal flood event	Multivariate	Tides, storm surges, and rivers inflows	**[N]**	Olbert et al.^23^
[2]	European year without a summer	Multivariate	Volcanic eruption and regional atmospheric circulation	**[N]**	Schurer et al.^24^
[3]	Ice storm	Temporally	Successive waves of freezing rain	Improving extreme weather forecasting **[S]**	Zhou et al.^25^
[4]	Wildfires	Multivariate	Thermal and vegetation stress	Improving wildfire forecasting driving anticipatory actions **[S]**	Nunes et al.^26^
[5]	Wildfires	Preconditioning	Antecedent drought, rare meteorology and human drivers	**[N]**	Ramos^27^
[6]	Crop failures	Multivariate	Hot and wet conditions	Improving forecasting of crop failures **[S]**	Ben-Ari et al.^28^
[7]	Ecosystem carbon uptake	Multivariate	Hot and dry conditions	Seasonal forecasting of summer extremes guided by interactions between carbon and water uptakes **[S]**	Bastos et al.^29^
[8]	Wildfires	Multivariate	Temperature, relative humidity, precipitation and wind speed	Improving wild fire forecasting **[S]**	Worsnop et al.,^30^ Di Giuseppe et al.^31^
[9]	Pluvial flood	Preconditioning	Rainfall on saturated soils	Improving seasonal forecasting of flood conditions for increased flood risk preparedness **[S]**	Bischiniotis et al.^18^
[10]	Simultaneous crop failures	Multivariate, Spatially	Hot and dry conditions in multiple growing regions	Improved impact forecasting **[S]**	Anderson et al.,^22^ Goulart et al.,^32^ Schroth et al.^33^
[E]	*Emergency response and civil protection*				
[1]	Landscape-scale wildfires	Spatially	Multiple large fires	**[N]**	Nolan et al.^34^
[2]	Wildfire management	Multivariate	Temperature, relative humidity, precipitation and wind speed	Sharing information on fire activity for emergency response units during fire events **[D]**	DaCamara et al.^35^
[3]	Arctic wildfires	Multivariate	Extreme drought and persistent heat waves	**[N]**	Irannezhad et al.^36^
[4]	Large floods in Europe	Spatially	Concomitant flood events across Europe	Input to international financial support schemes supporting anticipatory and recovery measures **[S]**	Jongman et al.,^37^
[5]	Tropical cyclone damage	Temporally	Consecutive tropical cyclones	Input to international financial support schemes supporting anticipatory and recovery measures **[D]**	Ciullo et al.^20^
[6]	Consecutive cyclone and heat wave	Multivariate	Combined cyclone and heat wave event	Supporting adaptation planning and recovery measures for disastrous tropical cyclones **[S]**	Matthews et al.^21^
[7]	Emergency evacuation due to dam damages	Temporally, preconditioning	Heavy rain on saturated soil and snowpack	Supporting design of flood response measures **[S]**	White et al.,^38^
[8]	Concurrent wildfire and heat stress	Multivariate	Heat stress and forest fires	Supporting allocation of emergency response resources under combined fire/heat stress conditions **[S]**	Vitolo et al.^39^
[I]	*Infrastructure risk management*				
[1]	Water reservoir failure	Multivariate	Human-induced water stress and climate change	Supporting design and management of water resource supply and demand **[S]**	Mehran et al.^40^
[2]	Coastal infrastructure failure	Temporally	Series of surge conditions	Supporting operational maintenance management of flood barrier **[S]**	Van Den Brink and De Goederen^41^
[3]	Spatially extensive infrastructure	Multivariate, temporally	Multiple meteorological hazards	Forecasting and management of electrical power shortfalls **[S]**	Turner et al.,^42^ Turner et al.^43^
[4]	Global supply chain	Multivariate	High precipitation due to monsoon and tropical cyclones	Supporting implementation of flood defences and flood management practices **[S]**	Haraguchi and Lall^44^ Gale and Saunders^45^
[S]	*Spatial planning and infrastructure design*				
[1]	Coastal flood event	Multivariate	Storm surge and heavy precipitation	Input to probabilistic risk assessment of coastal flood events supporting decisions on water management infrastructure **[S]**	Van Den Hurk et al.,^46^ Santos et al.^47^
[2]	Coastal flood event	Multivariate	Storm surge and high river discharge	Supporting risk assessment of coastal floods **[S]**	Bevacqua et al.,^19^ Khanal et al.^48^
[3]	Flood protection infrastructure	Multivariate	Storm surges and river discharges	Supporting evaluation of flood protection measures **[S]**	De Bruijn et al.^49^
[4]	Waves and sea level	Multivariate	Extreme coastal sea level	Supporting assessment of coastal infrastructure vulnerability **[S]**	Gouldby et al.^50^
[5]	Agricultural systems	Multivariate	Hot and dry conditions	Supporting design of agricultural adaptation measures **[S]**	Garry et al.,^51^ Feng et al.^52^
[6]	Energy networks	Spatially	Wind regimes across locations	Input to deployment strategies for wind energy **[S]**	Grams et al.^53^
[7]	Regulate spatial development	Multivariate, spatially	Multiple hazards and impacts affecting water management	Input to regional spatial planning policies **[S]**	De Smedt,^54^ Kabat et al.^55^
[8]	Cropland planning	Multivariate	Changes in temperature, rainfall, and evapotranspiration	Supporting crop spatial planning policies **[S]**	Schroth et al.^33^
[9]	Electrical power shortfall	Multivariate	Higher power demand and lower hydropower availability	Supporting design of shock-resistant energy infrastructure **[S]**	Turner et al.^42^
[C]	*Capacity building*				
[1]	Training material	Multivariate, temporally	Multivariate and consecutive hazards	Input to training material for DRR practitioners **[D]**	De Ruiter et al.,^56^
[2]	Attribution compound events	Multivariate, temporally	Attribution drivers of compound events	Input to public information on changing hazard probabilities **[S]**	van Oldenborgh et al.,^57^ Zscheischler and Lehner^58^

For each example an assignment of the appropriate compound event typology following Zscheischler et al, 2020,^10^ has been given together with a number of attributes of the governing compound features and application implementations as indicated in the case study paper. Labels in the column “Application” indicate whether a practical application is not mentioned (N), suggested (S), or demonstrated (D). Case study numbers and categories are referenced in Figure 1.

### Early warning systems and forecasting

In many cases, exceptional historic events trigger a “forensic” analysis of the drivers of the events. Examples of analyses of historic events are the 1816 European year without a summer, driven by a combination of a major volcanic eruption and a regional atmospheric circulation^24^; a large number of ice storms in China in 2008^25^; extensive coastal-fluvial flooding in Ireland due to exceptionally wet autumn in 2009^23^; an “Aqua Alta” compounding high water event in Venice^59^; and precursors of fire weather conditions in Portugal since 1980.^26^ For the wildfires in Portugal in October 2017—with nearly 200,000 ha of burnt area in just 24 h and 50 fatalities—several compounding features were playing a role.^27^ These included a long-term preconditioning leading to cumulative hydric stress of vegetation, the passage of hurricane Ophelia off the Coast of Portugal advecting large masses of hot and dry air, and a negligent human driver of ignitions associated with agricultural practices.

In the domain of early warning and forecasting the notion of compound drivers and hazards plays a dominant role. Combinations of multiple hazards that are not extreme by themselves can give rise to extreme impacts (e.g., Moftakhari et al., 2019^60^). Depicting the causal pathway from (multiple) hazards to (ultimately) the impacts requires the identification of metrics at the spatial and temporal scales at which the impact takes place.^13^ Once the relevant temporal and spatial scales are identified, forecasting approaches can be modified to better represent the required multivariable and spatial dependencies.^61^ In a generic perspective on the development of hydrometeorological forecasting^62^ argue that improved capability of monitoring and diagnosing forecasts of precursors of high-impact events does contribute to enhanced societal benefits of hydrometeorological forecasting services. New workflows to provide rapid access to forecast archives constrained by specific precursors are being set up.^63^

The development of impact-based forecasting is aided by a comprehensive assessment of the joint physical and statistical properties of the hydrometeorological impact-drivers. Impact-based forecasting is being developed in many areas, including power supply disruptions,^42^ tropical cyclone damage,^20^ crop failures,^28^^,^^32^ carbon uptake,^29^ wildfires,^30^^,^^31^ and their combination with heat stress.^39^ These forecasting systems require a thorough analysis of the multiple pathways that can lead to a pronounced societal or ecological impact, including the associated climatological distribution of compounding features that govern the impact occurrences. This requires not only a multi-disciplinary systems perspective but also a transdisciplinary approach where practitioners define critical thresholds^64^ and share experience on relevant usual (and unusual) process pathways.

### Emergency response and civil protection

High-impact emergency events are usually rare and trigger a multi-agency response from rescue services, security officers, construction builders securing access and limiting further damage and victims, health organizations, and crisis communication services. Usually these services consist of a mixture of institutionalized (government) organizations and ad hoc civil response units. These events also trigger the allocation of financial resources from public or private insurance or emergency recovery schemes. An effective emergency response to complex compound events overlapping in time and space is a challenge.^11^

Societal preparedness for hazardous conditions is promoted by a well-designed emergency response system, which is able to anticipate the frequency and spatial and temporal scales of events that cause major damage. The spatial extent of the 2019 Australian wildfires,^34^ for example, led to problems of fire escape routes. Well-designed information portals are being developed (e.g., for flood risk for Switzerland [https://www.hochwasserrisiko.ch/en], or the Ceasefire portal for wildfire management in Mediterranean Europe [https://www.ceasefire.pt/^35^]), but adequate monitoring is absent in remote areas such as those affected by Arctic wildfires.^36^ The awareness of cascading and compounding risks can also be used in training the people operating in the emergency response systems by constructing tailored event storylines.^65^ In addition, knowledge on the joint occurrence frequency of multiple crises within a given spatial and temporal domain governs the design of the emergency response infrastructure. This infrastructure should be sufficiently large, responsive, scalable, and mobile to contribute effectively to the societal preparedness for environmental risks.

### Infrastructure Risk Management

Large infrastructure systems, such as power plants including hydropower dams, power or mobility networks, flood protection infrastructure and many more, operate risk management protocols that minimize adverse impacts of environmental hazards. Risk management includes the routine operation of the infrastructure that takes variability and anticipation of extreme hydrometeorological conditions into account. It also encompasses emergency management during and after extreme events, including recovery and the resumption of key operational functions.

As an illustrative example, the California Oroville Dam in the Sierra Nevada experienced an unanticipated series of high-intensity Atmospheric River rainfall events in February 2017^38^ that led to the use of the emergency spillway and evacuation of a large community downstream of the dam. Water reservoirs often involve multiple functions, such as the combined use of reservoir water for hydropower and irrigation, or the contrasting interests of securing an ecological equilibrium ensuring flood protection in managed lakes.^66^ Together with the ongoing climatic trends, this multi-functionality poses constraints on the reservoir operation that is oriented at minimizing multi-faceted risk of failure to provide the required services.^40^ Risk management also refers to the maintenance protocols of the infrastructure, which is often governed by hydrometeorological seasonality, or can be triggered by preceding operation of the infrastructure. For instance, Van Den Brink and De Goederen (2017)^41^ explored the likelihood of recurrent closures of a flexible storm surge barrier. Tomasicchio et al. (2022)^67^ analyzed the compound statistics of high water levels in the Venice Lagoon supporting the operation of the MoSE barrier.

The standard operation protocols of these systems are designed to operate and monitor the infrastructure in a variable environment. They are usually designed to meet a number of contemporary demands, and thus are subject to trade-offs in a multi-criteria optimization procedure. Compound events in a constrained time window may impose large challenges to the infrastructure operation, particularly when conflicting demands (e.g., for safety, resource allocation, environmental protection, infrastructure maintenance) prevail. Spatially extensive infrastructure, such as a series of power plants, or widespread mobility or communication networks, require protocols that take temporally compounding events in the area of interest into account.^42^^,^^43^ Jongman et al. (2014)^37^ explored the degree to which correlated occurrence of floods within Europe may impair the European Union Solidarity Fund, finding the need to take climatic trends on the likelihood of compound flooding events into account. Ciullo et al. (2021)^20^ carried out a similar analysis of impacts of consecutive tropical cyclones. Knowledge on the compounding structure of occurrence of multiple drivers or hazards that induce emergency events helps the anticipation and (financial) recovery of these events.

Global supply chains are established through long and complex infrastructure, which are susceptible to disruptions due to compound events. An example often cited is the 2011 disruption of the supply chains of automotive and electronics industries, which were compromised due to heavy floods directly damaging industrial parks in Thailand.^44^ These floods were the result of high levels of precipitation due to an early summer monsoon from May to October combined with high precipitation from four tropical storms hitting the north of the country between June and October.^45^

In the agricultural sector, soybeans are consumed globally as they constitute the main source of protein for livestock feed worldwide, but are mostly produced in a limited number of key production regions in the United States, Brazil, and Argentina. Spatially compounding simultaneous weather anomalies across the main producing regions can lead to a shortage of soybeans in the global markets and record international prices.^32^

### Spatial planning and infrastructure design

The continuous development of the spatial configuration of inhabited areas provides many opportunities to increase future resilience against environmental impacts. Post-disaster recovery is seen as an essential ingredient of DRR frameworks.^15^ The land use and spatial planning imposed by developing economic exploitation, population growth, and associated infrastructure upgrades call for design principles that minimize the adverse impacts of hazards.^11^ Current and future patterns of compound high-impact events may govern design criteria of, e.g., flood protection infrastructure,^49^^,^^50^^,^^68^ agricultural systems,^51^^,^^52^ or renewable energy networks.^53^^,^^69^ It also informs policies to prohibit or regulate spatial development in risk-prone areas,^54^ or anticipate future climate conditions in the process of long-term spatial planning.^55^ Given the spatial variability of croplands, different spatial planning policies informed by future compound event risk can be considered, including changing cultivars or shifting croplands to regions with lower risk of impact-driving compound events.^33^

Extreme events may provide an incentive to reconsider or adjust the planning of large-scale infrastructure. For instance, a 2012 near-flooding event in a coastal region in the Netherlands^46^,^47^ was driven by a compounding surge and rainfall event leading to an extreme high water level in a coastal lake. The event fueled the design of pumping infrastructure to control this lake water level, and the likelihood of occurrence of a compounding surge and rainfall event drives the design criteria for this infrastructure. The analysis portrayed the univariate distribution of the lake level in which the multivariate characteristics of the surge and rainfall events convoluted, defined by the spatial and temporal scale at which these hazards combine into the impact indicator. Application of a similar combination of surge and rainfall to a larger European river system required different spatial and temporal aggregation to make a meaningful analysis of the role of the compounding hazards in governing the impact.^48^

Spatial planning and infrastructure design result from complex regulations and interactions between stakeholders and responsible organizations. The provision of information on (compound) hazard and impact statistics affects the public perception of the risks associated with compound events,^70^ and thus needs to be well tuned to the decision-making processes. A wide range of decision support tools and risk mapping platforms exist that are designed for this purpose.^13^^,^^71^ However, effective decision support also requires well-designed science/policy interactions tailored to the local decision contexts (e.g.,^3^).

### Capacity building

The literature on compound events analysis, classification, and interpretation is rapidly expanding, including case studies and review papers.^1^^,^^2^^,^^10^^,^^11^^,^^72^^,^^73^ This enhanced knowledge contributes to the development of novel concepts for mapping or analyzing compound event statistics. As such it helps the design and development of monitoring programs and forecasting systems relying on metrics that specifically express the risk features associated with compounding occurrence of impact-drivers. This insight also enriches the assessment of the climatological distribution of relevant compounding hazards and impacts by analysts of forecast or climate projection outputs.^62^^,^^63^

However, increasing the capacity to deal with or interpret compounding hazards by forecasters and practitioners in disaster risk management may contribute to public risk perception of high-impact events. Well-designed serious games or other training material can be useful to explore the dependence of hazard-impact pathways on compounding drivers, including factors affecting the societal or ecological exposure and vulnerability.^56^ This requires a multi- and transdisciplinary approach, binding physical hazards, societal or ecological impacts, and statistical descriptions. Attribution of extreme events governed by compounding drivers to anthropogenic climate change can contribute to general awareness of their role in shaping high-impact events, but requires a careful analytical framework to arrive at reliable conclusions.^57^^,^^58^

## Discussion and conclusions: Ways forward in “compound thinking” from an application perspective

Each of the examples listed above under the different disaster risk reduction application categories can be associated with one or more of the compound event types identified by Zscheischler et al. (2020)^10^ (see Table 1). Also included is a brief description of the applications whose implementation requires the consideration of relevant compound features. The applications listed are suggested by the cited references, and in some occasions also implemented and evaluated. Some studies did not refer to a potential application, but addressed compounding event features that may be relevant for the application categories described above. This listing intends to inspire practitioners to improve the management of environmental risks.

In each of the disaster risk reduction application classes discussed here multiple types of compound events can be found. The application domains in each of the classes are affected by compounding impact-drivers that require a thorough understanding of the interacting processes leading to societal or ecological impacts. In the forecasting and early warning systems and their underlying statistical climatologies, complex causal event cascades require consideration of multiple ancillary factors determining the magnitude or extent of the impact. Verification of statistical or physical models needs to be guided by definition and selection of appropriate diagnostic metrics, conditioned on the compounding drivers, hazards, or impacts. For emergency response and civil protection, “compound thinking” incorporates the build-up of experience with disproportional impacts of consecutive or spatially compounding events, which affect the way resources are optimally distributed over the various response actions. Management of large or widely distributed critical infrastructure also requires understanding of complex impact cascades, feeding into appropriate management strategies and stress testing criteria to optimize the functioning of the infrastructure. The design of spatial planning or protection infrastructure could utilize evidence-based assessments of multiple hazards challenging the region of interest in multiple directions. And adopting continued learning cycles, for instance, by frequent assessments of real-world or simulated events, can contribute to public awareness and operational coping capacity.

Similar to risk assessment procedures based on univariate hazard-impact cascades, compound events generate impacts by operating at a specific temporal or spatial scale. The required statistical description of compounding features needs to result in an improved representation of the resulting impacts, and this requires a proper analysis of the appropriate temporal averaging and/or temporal delays of compounding drivers or hazards, and the spatial dimension where the drivers and hazards coincide and result in an impact. Flood risks are strongly governed by the topography of river and coastal systems, and the spatial and temporal aggregation of the impact-drivers needs to correspond with the size and response timescale of these basins and coastal areas. The timescales at which preconditioning compounding features can have an impact depends on the “memory” of the prior conditions, which may be linked to the seasonal cycle^28^ or spatial scale of the domain of interest.^74^

The inspiration to incorporate compounding impact-drivers across the application domains is very often delivered by evidence from real-world events, either at the location of interest or in remote regions. A systematic analysis of all potential compounding impact-drivers, particularly those features that have no precedent in the observational records, is extremely challenging, if not impossible. However, historic events may be combined with exploratory hypothetical “counterfactuals,”^75^^,^^76^ by analyzing impacts of smaller or larger perturbations to the historic event. Literature on “event storylines” is emerging^65^^,^^77^ and illustrates avenues to explore in the future. This includes unprecedented compounding impact-drivers, or the way a historic event could evolve under future climatic or societal conditions.

The broad domain of the practice of risk management encompasses both the implementation of DRR principles in present-day policies and the exploration of trends in risks (and their management options) under a range of future climatic or societal conditions. However, in this article we have not made an explicit distinction between present-day and future application domains. In practice there is a large overlap in concepts, policies, and responsible entities for current and future risk management. And the role of compounding impact-drivers is in principle equivalent for these time windows, albeit that climate change and societal development do impose trends on both compounding drivers and their potential impacts.^2^

The scientific analysis of compounding impact-drivers is accelerated by adopting an impact-centric perspective. The cascading event chain that links drivers, hazards, and impacts is primarily meaningful for societal applications when the implications for societal impacts are emphasized. The analytical framework of compounding events requires a selection of hazard and driver attributes that coincide to affect the potential impacts, and this selection includes an appropriate definition of spatial/temporal dimensions, physical attributes, and statistical thresholds.

Simultaneously, the observational evidence base of compounding impact-drivers does require a careful documentation of impacts and their (compounding) causes. Impact databases such as EM-DAT^78^ are often used for analyses of trends, drivers, or forecasting of environmental disaster impacts. However, a systematic registration of impact-drivers in these databases is not present yet, and further work in this direction is desirable.^79^

Considerable added value can be expected from scientific analysis frameworks that enable the exploration of future risks imposed by unprecedented compound event cascades. A diversity of conceptual analysis frameworks—including statistical models,^13^ dynamical modeling,^46^ counterfactual analysis,^77^ climate storylines,^80^ and others—can lead to practical tools to generate compound event scenarios that can guide design standards or improve adaptive capacity. Also the definition of appropriate compound metrics is required, to support monitoring and definition of model output diagnostics that support the assessment of the role of compound events in risk profiles.

The practice of scientific analysis of compounding impact-drivers strongly relies on a multi- and transdisciplinary team configuration. Inputs from practitioners and a wide diversity of scientific disciplines, including hydrometeorological hazards, statistics, ecological processes, societal impacts, and the design of effective decision support tools, are required to explore and further develop the application domains presented here.
